# Discerning two-dimensional metal halide perovskite moieties using solid-state NMR fingerprints

**DOI:** 10.1039/d5ta02747k

**Published:** 2025-08-05

**Authors:** Federico Brivio, Nurgul Sarsembek, Giulia Martelli, Rolando Scotillo, Fabio Loprete, Filippo De Angelis, Daniele Cortecchia

**Affiliations:** a Department of Physics and Astronomy “Galileo Galilei”, University of Padova Via Francesco Marzolo 8 35131 Padova Italy Federico.Brivio@unipd.it; b Department of Chemistry, Biology and Biotechnology, University of Perugia, Computational Laboratory for Hybrid/Organic Photovoltaics (CLHYO), Istituto CNR di Scienze e Tecnologie Chimiche “Giulio Natta” (CNR-SCITEC) Via Elce di Sotto 8 06123 Perugia Italy; c Department of Industrial Chemistry “Toso Montanari”, University of Bologna Via Piero Gobetti 85 40129 Bologna Italy Daniele.Cortecchia2@unibo.it; d Institute of Energy Science and Technology (SIEST), Sungkyunkwan University (SKKU) Suwon 440-746 South Korea; e Consorzio Interuniversitario Nazionale per la Scienza e Tecnologia dei Materiali (INSTM) – Unità di Ricerca (UdR) di Perugia Via Giuseppe Giusti, 9 50121 Firenze Italy; f Center for Nanoscience and Technology, Istituto Italiano di Tecnologia Via Rubattino 81 20134 Milano Italy

## Abstract

Low-dimensional perovskites hold great promise for optoelectronic applications, spanning from photovoltaics to photonic platforms. The electronic properties of these soft hybrid semiconductors critically depend on their crystal structure, and the ability to clearly discern the connectivity of the inorganic component and the organic molecular packing is crucial to foster the development of improved materials. In this work, we apply solid-state NMR spectroscopy (ssNMR) to compare two perovskites with different structural motifs: the flat 〈100〉-oriented Ruddlesden–Popper (BA)_2_PbI_4_ and the corrugated 〈110〉-oriented Dion–Jacobson phase (EDBE)PbI_4_, which are flat and corrugated layered perovskites, respectively. Combining experimental characterizations under static and magic angle spinning conditions up to 42 kHz with spin relaxation time measurements and density functional theory (DFT) calculations, we show that ^13^C, ^15^N and ^207^Pb spectroscopies provide distinct spectral fingerprints that are characteristic of the perovskites’ different connectivities and allow the identification of their local spatial arrangements. Our work provides a deeper understanding of the ssNMR response of 2D perovskites with different connectivities of their inorganic framework, highlighting its key role as a complementary technique to traditional diffraction-based methods.

## Abstract 2

Solid-state NMR spectroscopy allows clear differentiation of the local spatial arrangements at the supramolecular level in 〈100〉- and 〈110〉-oriented layered perovskites, which arise from the characteristic connectivity of their inorganic frameworks.

## Graphical abstract

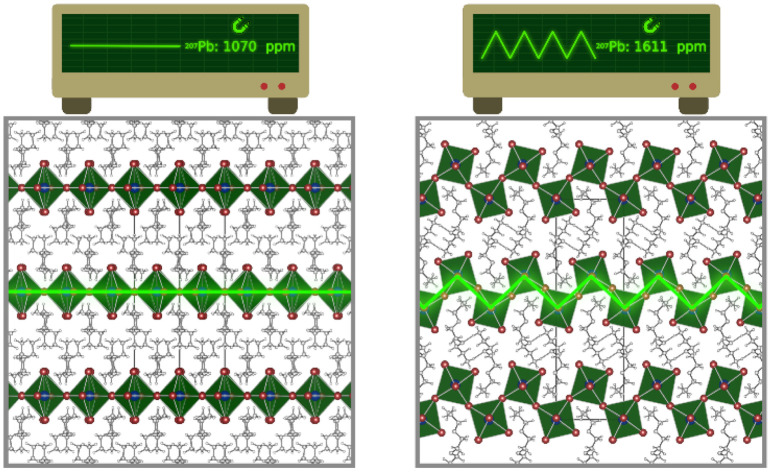

## 1 Introduction

**Fig. 1:**
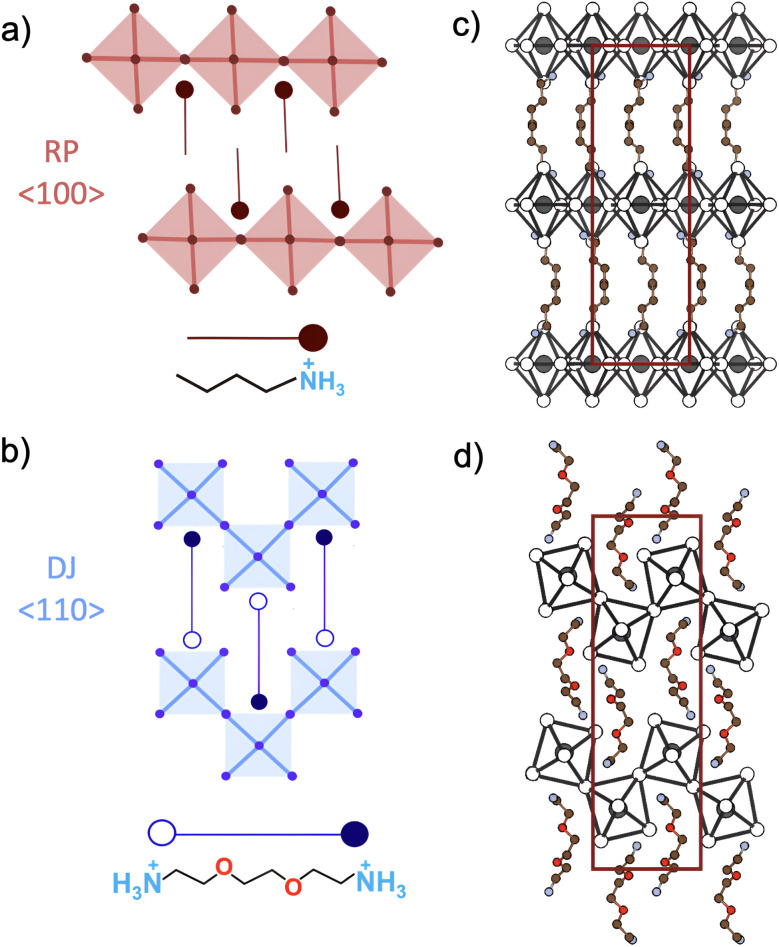
Schematic representation of the flat 〈100〉-oriented Ruddlesden–Popper perovskite templated by BA^+^ cations (a), and of the corrugated structure of the 〈110〉-oriented Dion–Jacobson perovskite templated by EDBE^2+^ cations (b); open and filled circles in the schematic ligand represent ammonium moieties with different coordination environments located at inequivalent crystallographic sites. The red and blue squares represent the individual PbI_6_^4−^ octahedral units of the inorganic layers. (c) Crystal structure of BA_2_PbI_4_ with space group *Pbca* (no. 61). (d) Crystal structure of (EDBE)PbI_4_ with space group *P*2_1_/*c* (no. 14). Both structures’ *b*-axis has been aligned in the *x*-direction. The unit cells are highlighted in red, while H atoms have been omitted.

Low-dimensional metal halide perovskites (MHPs) are attracting great interest for applications in photovoltaics and photonics.^1,2^ Functional organic cations are being explored as templating units to obtain perovskites with new photophysical properties, improved efficiency and enhanced stability.^3,4^ This enables many synthetic possibilities due to the richness of dimensionalities and coordination environments, which are critical in determining the final properties of the material. With the increasing chemical complexity, however, standard X-ray diffraction (XRD) methods are becoming insufficient for capturing a complete picture of the perovskite structure. Factors such as poor crystal quality,^3,5^ dynamic disorder^6–10^ and the presence of amorphous domains can prevent the satisfactory solution of the superlattice structure.^11,12^ In fact, while several metal halides with functional cations have been reported and spectroscopically characterized, the structures of many of them have not been solved *via* single-crystal diffraction techniques^3^ leaving the perovskite formation uncertain in several cases.^13–16^ This is a huge limitation towards the understanding of the structure–properties relationship and the development of rational crystal engineering strategies. Solid-state nuclear magnetic resonance (ssNMR) is an alternative technique that can help overcome these limitations.^17^ Solid-state NMR has recently been applied to the investigation of 3D perovskites and molecular additives for defect passivation.^7,18–22^ However, only a few works have focused on the study of layered perovskites,^23–26^ particularly concerning the local structural features of perovskites with different dimensionalities of the Ruddlesden–Popper (RP) monovalent organic cation series.^6,27–29^ When monovalent cations are substituted with divalent cations, layered perovskites are obtained where adjacent inorganic layers are directly linked by a single organic molecule without the presence of a van der Waals gap (Fig. 1). These have been classified by some authors as Dion–Jacobson (DJ) perovskites based on the ditopic nature of the templating cations.^3,30,31^ However, another classification, to which we will refer, considers the crystallographic properties and the relative alignment between adjacent inorganic layers, which adopt a staggered or eclipsed geometry in RP and DJ phases, respectively.^32–34^

Depending on the choice of the cations, different connectivities of metal halide octahedra may be achieved, which can be seen as derived by slicing the parental 3D perovskite lattice along different crystallographic planes. Typical examples are the 〈100〉-oriented and 〈110〉-oriented 2D perovskites that assume a flat motif and a corrugated motif of the inorganic framework, respectively.^35^ The ability to distinguish between the different types of connectivity is crucial because it deeply affects the optoelectronic properties.^36^ Nevertheless, the use of ssNMR to distinguish the two coordination environments has not been deeply investigated. Moreover, the interpretation of the ssNMR response is not always straightforward, as complicated lineshapes and spectral responses with multiple peaks might hamper a correct interpretation. Density Functional Theory (DFT) calculations can provide valuable complementary information to the experimental data but, surprisingly, only a few works have been dedicated to the prediction of the NMR response in metal halide perovskites. These include the investigation of halide occupational positions in (BA)_2_PbX_4_,^37^ but mostly employing a finite cluster model.^38–41^ The NMR response of cluster models can be calculated by including full spin–orbit coupling (SOC) correction, but this requires the construction of a model for each atom studied. While SOC correction has not been fully included in the DFT codes that rely on the Gauge Including Projector Augmented Waves (GIPAW) theory, models based on periodic boundary conditions require a unique structure to obtain the NMR response of all the atoms. This come at the cost of possible inclusion of systematic errors, but considers a more representative structure of the material.^42,43^

For these reasons, more efforts shall be dedicated to the development of a reliable computational approach to support the experimental findings and assist the structural elucidation of low-dimensional perovskites. In this work, we consider as model compounds with different connectivities of the inorganic framework the two RP layered perovskites (BA)_2_PbI_4_ (Fig. 1a and c) and (EDBE)PbI_4_ (Fig. 1b and d) based on *n*-butylammonium (BA) and 2,2-(ethylenedioxy)bis(ethylammonium) (EDBE) cations.

The first crystal is a 〈100〉-oriented RP perovskite, with orthorhombic lattice and space group *Pbca* (no. 61),^44^ while the second is a 〈110〉-oriented DJ perovskite with a monoclinic cell and space group *P*2_1_/*c* (no. 14).^45^

The 〈110〉-orientation is a relatively rare coordination for lead iodide perovskites, which has been identified in only a few compounds, like (IFO)_2_PbI_4_ (IFO = iodoformamidinium),^46^ (C(NH_2_)_3_)_2_PbI_4_,^47^ (3APr)PbI_4_ (3APr = 3-aminopyrrolidinium)^48^ and (EDBE)PbI_4_. Among these, the latter was taken as an archetypal compound since it allows templating along the 〈110〉-orientation while minimizing the differences in the organic components with (BA)_2_PbI_4_, since they are both based on linear cations and EDBE^2+^ has approximately double the length of BA^+^.

We show that the flat and corrugated structural motifs of these two perovskites are characterized by markedly different ssNMR responses of ^13^C, ^15^N and ^207^Pb nuclei. By combining experimental data with theoretical calculations employing the Gauge Including Projector Augmented Waves (GIPAW) method within the density functional theory (DFT) framework,^49–51^ we are able to fully elucidate the spectral assignment in relation to the local coordination environments. In the organic part, differences in chemical shift and peak splitting for ^13^C and ^15^N nuclei in (EDBE)PbI_4_ arise from the symmetry-induced inequivalence of crystallographic sites with different supramolecular arrangements characteristic of the 〈110〉-oriented perovskite. Meanwhile, comparing ^207^Pb spectra under static and magic angle spinning conditions up to 42 kHz, we show that ^207^Pb can be conveniently used as a structural probe of the inorganic component,^52^ as it displays a 541 ppm shift at higher frequency going from (BA)_2_PbI_4_ to (EDBE)PbI_4_, in addition to markedly higher values of chemical shift anisotropy and asymmetry parameters for (EDBE)PbI_4_, correlating with the increased distortion of its octahedral coordination. This work deepens the understanding of the ssNMR fingerprints of layered perovskites, laying an experimental and theoretical background that will foster its application to more complex structures and enable new synthetic approaches towards more functional structures.

## 2 Results and discussion

### 2.1 Structural properties

(BA)_2_PbI_4_ and (EDBE)PbI_4_ were synthesized from concentrated water-based HI solutions by slow cooling and antisolvent diffusion methods, respectively. After filtration and vacuum drying, the crystals were finely ground for subsequent characterizations, and the achievement of the desired perovskite phases was confirmed by powder XRD (see Fig. S1).

Fig. 1c and d report the two unit cells and highlight the organic linkers for each. The inorganic part of the (BA)_2_PbI_4_ structure is formed by four identical corner-sharing PbI_6_ octahedra that span the *ab*-plane, with both the two apical undercoordinated I atoms pointing along the *c*-axis, endowing a flat arrangement of the inorganic sheets. These undercoordinated I atoms are opposite to each other with respect to the Pb atom, and therefore form a 180° angle with it. In the unit cell, consecutive PbI_6_ planes are obtained by shifting along the vector 
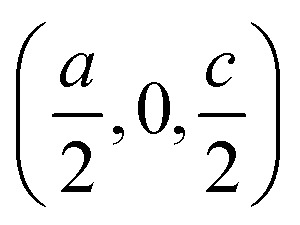
 in agreement with the space group symmetry. The adjacent inorganic layers have a perfect staggered alignment typical of Ruddlesden–Popper perovskites (schematic representation in Fig. 1a).^32–34^ The inorganic sheets are connected by four organic CH_3_(CH_2_)_3_NH_3_^+^ cations arranged in tail-to-tail bilayers with the respective –NH_3_^+^ groups pointing toward the PbI_6_ groups. The –NH_3_^+^ groups are located closer to the octahedra pocket, thereby maximizing the interaction with the I atoms.

The (EDBE)PbI_4_ structure exhibits more distorted PbI_6_ octahedra (see Table S1) with a different corner-sharing connectivity. The undercoordinated I atoms of consecutive octahedra point in opposite directions, leaving the two undercoordinated I atoms of the same octahedra to be adjacent and therefore forming an almost perfect right angle with the Pb atom.

Small shifts of 0.28 Å and 0.04 Å in the *a*- and *c*-directions, respectively, between adjacent inorganic planes causes a slight off-centering in the stack of the octahedra, which nevertheless adopt a nearly eclipsed configuration characteristic of the DJ phases (schematic representation in Fig. 1b).^32–34^

The EDBE organic cations are aligned in the *b*-direction. One of the –NH_3_^+^ groups is located in the pocket defined by the octahedra, while the other forms H-bonds with the ether group of the adjacent molecule and closely interacts with a single octahedral unit. This supramolecular interaction between adjacent EDBE cations is responsible for the zig-zag arrangement of the inorganic framework and further induces a considerable octahedral distortion of the lead environment. With respect to (BA)_2_PbI_4_, this reflects the different interactions between the NH_3_^+^–PbI_6_^4−^ units in the two models.

### 2.2 ^13^C and ^15^N NMR response

The carbon and nitrogen environments were studied using cross-polarization magic angle spinning (CPMAS) spectroscopy, which involves the excitation of abundant ^1^H nuclei followed by magnetization transfer to the ^13^C and ^15^N nuclei mediated by dipole–dipole coupling, allowing an enhancement of the signal intensities of the low-abundance ^13^C and ^15^N nuclei.

**Fig. 2:**
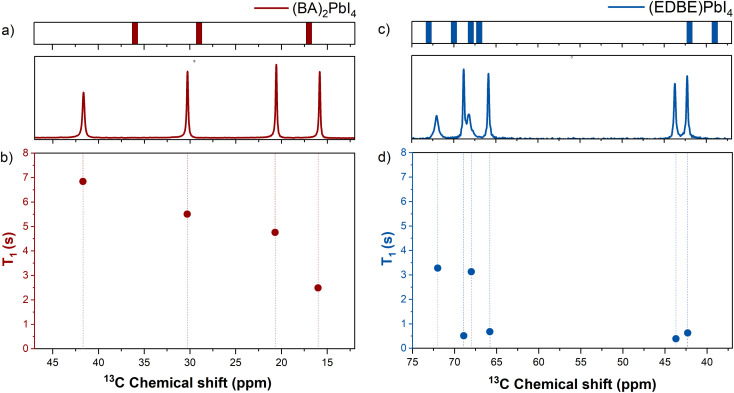
^13^C ssNMR (CP-MAS) spectra for (BA)_2_PbI_4_ (a and b) and (EDBE)PbI_4_ (c and d). The top panels in figures (a and b) represent the chemical shifts computed using DFT (vertical bars), while the bottom panels show the corresponding experimental spectra. The different crystal symmetries result in a different number of peaks for each species. In the ^13^C spectra it is possible to distinguish 4 different peaks for (BA)_2_PbI_4_ and 6 for (EDBE)PbI_4_. The DFT value of around 17 ppm for the C atom in (BA)_2_PbI_4_ corresponds to two overlapping signals with the same isotropic chemical shift. Figures (c and d) show the longitudinal spin–lattice relaxation times *T*_1_ as a function of the chemical shift (see Tables S7 and S8).

In (BA)_2_PbI_4_, the butylammonium cations are all equivalent, and therefore four ^13^C signals are observed as expected. The most upfield signal belongs to the –CH_3_ group and the others to the progressively deshielded methylene groups, as reported in the bottom panel of Fig. 2a, in agreement with previous reports.^6,25^

In light of its symmetry, three ^13^C signals are expected for EDBE. However, the formation of two crystallographically (and magnetically) independent sites in the asymmetric unit (Fig. 1b and d) implies that the resulting spectroscopic signals reflect both the chemical nature and the crystallographic position within the cell.^53^ Correspondingly, we observe a split of the signal into 3 couples of peaks, as shown in the bottom panel of Fig. 2c.

Further insights were obtained through measurements of the spin relaxation time (Fig. 2b and d, and Tables S3 and S4). Since the spin relaxation time of ^13^C nuclei is influenced by the dipolar coupling to ^1^H and by the locally oscillating magnetic fields, this can be used to assess the molecular motion at each specific position of the molecule.^6^ Specifically, the spin–lattice relaxation time *T*_1_ probes fast molecular motions occurring close to the Larmor frequency (MHz range).^52,54,55^ However, it should be noted that direct correlations between molecular motions and spin–lattice relaxation times are not straightforward, as *T*_1_ values do not follow a monotonic trend with the correlation time of motion and are further influenced by the number of protons attached to each specific carbon.^54,56^

In (BA)_2_PbI_4_, we find that *T*_1_ progressively decreases from 7 s to 2.5 s moving towards the most shielded peak (Fig. 2b); this trend is consistent with the one reported by Landi *et al.*^6^ This is also reflected in the intensity build-up evolution of the cross-polarization ^13^C{^1^H} CP-MAS spectra measured at different CP contact times (Fig. S2), where the magnetization transfer rate from the protons to the ^13^C nuclei gets progressively smaller moving from the CH_2_ group near the ammonium moiety (6.19 ms^−1^) towards the terminal methyl group (0.55 ms^−1^). The –CH_3_ group experiences the slowest CP build-up compared to the methylene groups due to the rapid molecular rotation along its axis, reducing dipolar interactions between its component spins.^57^ We associate these trends to the very rapid rotation of the terminal methyl group in agreement with previous reports.^6,25^ On the other hand, we find generally faster relaxation times for (EDBE)PbI_4_ with *T*_1_ ranging from 0.4 s to 4.0 s (Fig. 2d). Here, the peaks at 72 and 68 ppm show a considerably longer *T*_1_ compared to the rest of the chain. As these signals correspond to the carbons of the central ethylene unit (see below for the full assignment), we attribute their slower decay dynamics to the involvement of the nearby ether groups in the intermolecular hydrogen bonding with the ammonium moieties of the adjacent EDBE cations (Fig. S3).

To substantiate our analysis, we further measured the ^1^H spectra and *T*_1_ relaxation times (Fig. S4). At 42 kHz MAS, the spinning rate partially averages the strong ^1^H–^1^H dipolar couplings, enabling limited peak resolution while preserving efficient spin–lattice relaxation. Notably, the ^1^H *T*_1_ values across all resonances were nearly identical (see Tables S3 and S4), consistent with the behaviour observed in proton-dense solids, where extensive ^1^H–^1^H dipolar networks and fast spin diffusion equilibrate relaxation throughout the system.^58^ However, shorter ^1^H *T*_1_ times were observed for (EDBE)PbI_4_ (average *T*_1_ = 2.03 s) compared to (BA)_2_PbI_4_ (average *T*_1_ = 3.83 s), in agreement with the observations based on the ^13^C *T*_1_ analysis.

We further supported the structural characterization by directly probing the ammonium moieties of the two perovskites. ^14^N and ^15^N measurements have been previously adopted in a few works to probe the coordination of cations and molecular passivators in the perovskite structure. ^14^N NMR strongly relies on the analysis of the spinning sideband pattern, but quadrupolar broadening and spectral complexity can limit its application.^21,59–61^ Here we show that ^15^N spectroscopy can distinguish with great accuracy the different local coordination environments of 2D perovskites both in terms of spectral shift and spin–lattice relaxation dynamics, providing a clear fingerprint of the presence of independent crystallographic sites with different coordination chemistries in 〈100〉- and 〈110〉-oriented 2D perovskites.

**Fig. 3:**
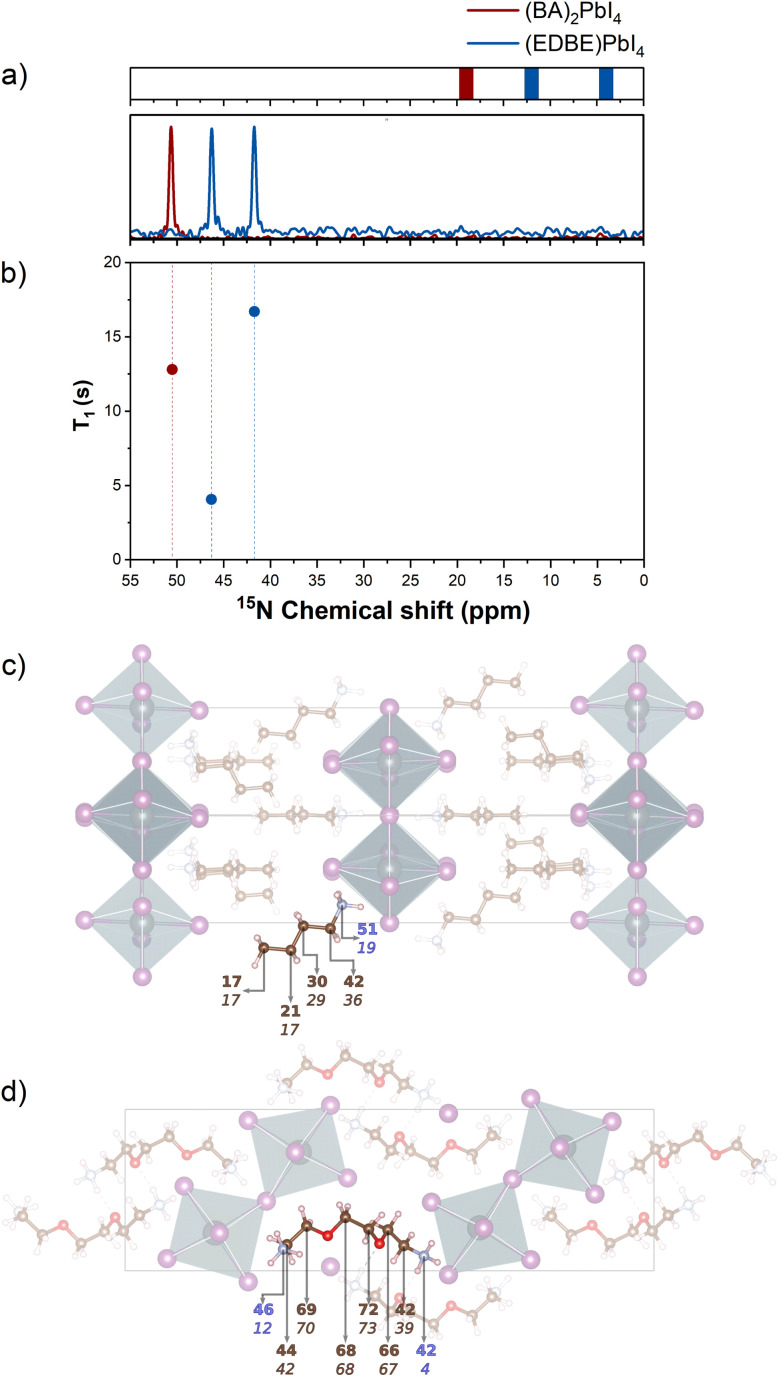
(a) ^15^N ssNMR (CP-MAS) response for (BA)_2_PbI_4_ (dark red) and (EDBE)PbI_4_ (blue) corresponding spin–lattice relaxation times (panel b). The ^15^N spectrum of (EDBE)PbI_4_ shows two peaks due to the presence of symmetrically nonequivalent N atoms. The predicted chemical shifts for the N atoms present a systematic error of roughly −30 ppm. The chemical shifts have been obtained from the chemical shielding as explained in the SI (see Tables S7 and S8). (b and c) Highlight of the molecular linkers in (BA)_2_PbI_4_ (c) and (EDBE)PbI_4_ (d). The top bold labels report the experimental chemical shifts of C (brown) and N (blue) atoms in ppm, while the lower italic numbers refer to the computed ones. In the schematic representation of Fig. 1b, the nitrogen atom with the highest shielding is represented by an open circle, while the other is represented by a full circle.

In the (BA)_2_PbI_4_ structure, there is a single crystallographically independent N atom that forms a distorted pyramid with the I atoms, with a corresponding single peak at 51 ppm and relaxation time *T*_1_ of 12.8 s (dark red peak in Fig. 3a). One of the N atoms of (EDBE)PbI_4_ (open circle site in Fig. 1b) forms an analogous pyramidal structure with the same N–I bond length of 3.60 Å. The distortion of their coordination environment can be quantified considering the distortion index parameter (see Table S2), corresponding to 0.0358 for (BA)_2_PbI_4_ and 0.0275 for (EDBE)PbI_4_ (nitrogen site represented with an open circle in Fig. 1b). Due to the resulting different local environment, the NMR signal of this ^15^N site of (EDBE)PbI_4_ shifts to 42 ppm, compared to 51 ppm in (BA)_2_PbI_4_, and exhibits a longer relaxation time of 16.7 s. The other (EDBE)PbI_4_ ammonium moiety (filled circle site in Fig. 1b) wedges between the PbI_6_ octahedra forming three bonds with the I atom and has an intermediate chemical shift value of 46 ppm with a significantly shorter *T*_1_ of 4 s.

More generally, we can conclude that the increasingly distorted environment contributes to the observed deshielding of ^15^N nuclei, and it is supported by DFT assignment as shown in the top panel of Fig. 3a. In particular, for the C species, the theoretical predictions are in good agreement with the experimental data as evident from the top panels of Fig. 2a and c; they differ at most by 6 ppm for the C closest to the BA–NH_3_^+^ group, but with an average error of only 1 ppm. DFT calculations can also provide the full chemical shielding tensor, allowing the analysis of anisotropy and asymmetry (see Tables S5 and S6).

The same agreement is not found for the ^15^N signal, which is underestimated by roughly 30 ppm. This discrepancy could be due to a poor description of the local environment,^62^ or, more likely, to the vicinity to Pb atoms, which are described with just a partial SOC correction. A similar effect has been reported in the literature for a system containing N and Br.^63^ Nevertheless, in light of the good correlation between experimental chemical shifts and theoretical chemical shielding (*m* = −0.973, *r*^2^ = 0.994, see Table S7), the relative difference between different N atoms is well reproduced. The two experimental peaks in (EDBE)PbI_4_ are separated by 8 ppm, while the corresponding theoretical ones are separated by 6 ppm. The lowest EDBE peaks are separated from the BA one by 9 ppm in the experiment and 15 ppm in the simulations.

### 2.3 ^207^Pb NMR response

^207^Pb is a heavy element with the highest atomic number among the spin-½ nuclei, making its NMR response highly sensitive to variations in its local electronic environment. In solid-state systems, the spatial distribution of electron density around Pb is often anisotropic, meaning that the nucleus experiences a different chemical shielding tensor depending on its orientation relative to the external magnetic field. This phenomenon, known as chemical shift anisotropy (CSA), provides critical information about electronic asymmetry, bonding interactions, and structural distortions, particularly in probing Pb–I coordination, allowing for a comparative analysis of (BA)_2_PbI_4_ and (EDBE)PbI_4_.

Differences in coordination geometry and electronic distribution result in distinct CSA parameters, and the chemical shielding tensor (*σ*) quantifies how the local magnetic field at the nucleus deviates from the applied field (**B**_0_), expressed as 
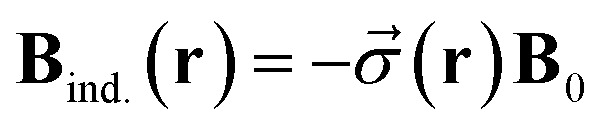
. In the principal axis system, the tensor components (*σ*_*xx*_, *σ*_*yy*_, *σ*_*zz*_) define the isotropic chemical shielding (*σ*_iso_), and the shielding anisotropy (Δ*σ*), which is a measure of the largest deviation in chemical shift from the average (isotropic) value. The chemical shift asymmetry parameter (*η*) describes the deviation from axial symmetry, providing further insight into distortions in Pb coordination, given by 
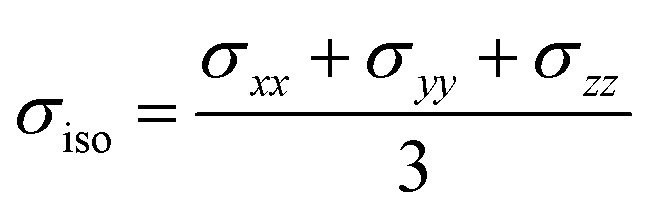
; Δ*σ* = *σ*_*zz*_ − *σ*_iso_; and 
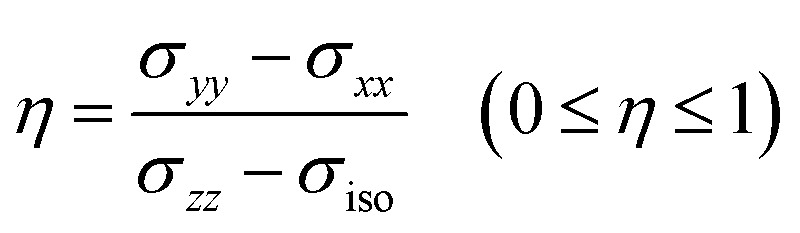
, respectively.^64^

**Fig. 4:**
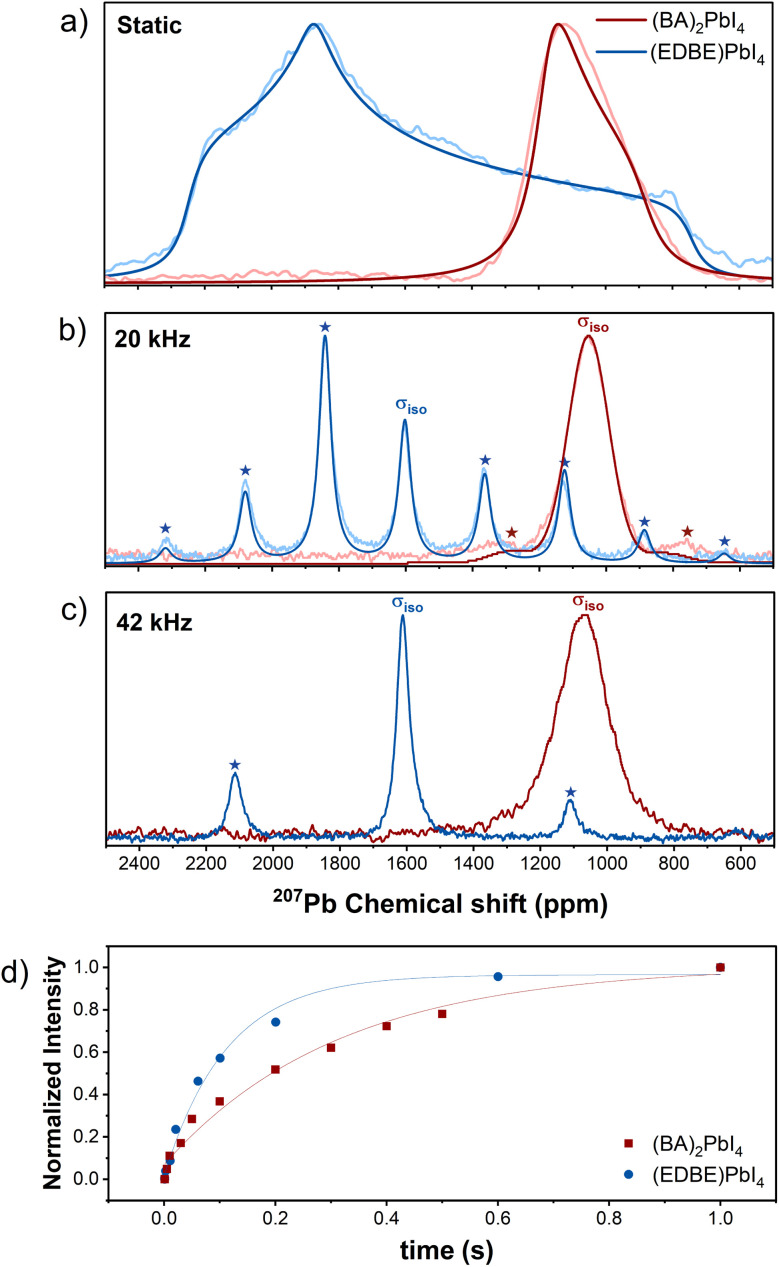
Experimental ^207^Pb ssNMR (Hahn-echo) spectra and corresponding fit for (BA)_2_PbI_4_ (dark red) and (EDBE)PbI_4_ (blue) under different conditions: (a) static rotor (*T* = 298 K); (b) 20 kHz MAS rate (*T* = 298 K); (c) 42 kHz MAS rate (*T* = 315 K). The reported temperature is the actual temperature of the sample by taking into account the contribution from frictional heating. The isotropic chemical shift is marked as *σ*_iso_, while the other peaks indicated by stars are side bands arising from the rotor frequency. We can observe how the proportion of the distances at different rotation speeds correspond to the rotor frequency. Data relative to other rotor speeds are included in Fig. S6. (d) ^207^Pb spin–lattice relaxation dynamics from saturation recovery measurements under static conditions.

The static ^207^Pb NMR measurements shown in Fig. 4a provide insights into the full CSA tensor, capturing the orientation-dependent interactions that would be averaged out under MAS conditions. Fitting of the experimental data was performed following the Haeberlen convention |*σ*_*zz*_ − *σ*_iso_| ≥ |*σ*_*xx*_ − *σ*_iso_| ≥ |*σ*_*yy*_ − *σ*_iso_| to retrieve the full chemical shift principal tensor values.^65,66^ For (BA)_2_PbI_4_, static measurements reveal an isotropic shift of 1076.8 ppm, a Δ*σ* value of −196.36 ppm, and an asymmetry parameter *η* = 0. This is in excellent agreement with the data reported by J. Lee *et al.* (Δ*σ* = −207 ppm; *η* = 0)^28^ and by Hope *et al.* (Δ*σ* = −270 ppm; *η* = 0),^37^ while Landi *et al.* reported a higher CSA value (Δ*σ* = −410 ppm; *η* = 0)^6^ for the same material. In contrast, in (EDBE)PbI_4_ we find a significantly larger anisotropy, with *σ*_iso_ = 1603.26 ppm, Δ*σ* = −855.93 ppm, and *η* = 0.422, together with a broader and more deshielded signal. This correlates well with previous works showing that ^207^Pb spectroscopy allows the true chemical speciation of the lead nucleus in metal halide perovskites to be probed.^67^ Due to the lack of resolution in static measurements and the tensorial nature of the NMR interaction, we further employed MAS, as also demonstrated by Hanrahan *et al.*^68^ to be highly effective in enhancing both resolution and sensitivity in lead halide perovskites, removing anisotropic broadening and enabling a more precise determination of the isotropic shifts. At 20 kHz MAS frequency (Fig. 4b), (EDBE)PbI_4_ still shows the isotropic signal flanked by a complex spinning sidebands manifold, in contrast to (BA)_2_PbI_4_ where the same rotation frequency is sufficient to almost completely spin out the chemical shift anisotropy interactions leading to the presence of just two weak sidebands.^69,70^ Fittings of the sideband patterns confirmed the results obtained from the lineshape analysis of static measurements, resulting in Δ*σ* = −813 ppm and *η* = 0.41 for (EDBE)PbI_4_, and Δ*σ* = −183 ppm and *η* = 0 for (BA)_2_PbI_4_. The small asymmetry and anisotropy of the chemical shift tensor of (BA)_2_PbI_4_ is consistent with the nearly cubic symmetry of the iodine atoms surrounding the lead nuclei with a close to ideal octahedral coordination.^71^ This is in sharp contrast with the case of (EDBE)PbI_4_, where the higher CSA and *η* are consistent with stronger anisotropic interactions reflecting pronounced distortions in the Pb coordination geometry (see Table S1).^36,72^ A fast MAS frequency of 42 kHz allows high resolution spectra to be obtained, increasing the intensity of the central peak in particular for EDBE and highlighting the difference in the isotropic chemical shift *σ*_iso_ centered at 1611 ppm and 1070 ppm for the (EDBE)PbI_4_ and (BA)_2_PbI_4_ perovskites, respectively. This 541 ppm difference is indicative of a considerable deshielding of ^207^Pb nuclei from electron density induced by the tilted structural arrangement. In this case, we did not perform a full lineshape analysis since the lack of a complete sideband manifold hampers a quantitative extraction of the tensor parameters.^70,73^ We note that the exact peak position will depend on temperature, with a variations of 1.1 and 0.6 ppm K^−1^ for (BA)_2_PbI_4_ and (EDBE)PbI_4_ within our probed temperature range (see Fig. S5). We attribute this difference in the peak shift rate with temperature to the easier structural reorganization allowed by the presence of the van der Waals gap in the RP perovskite phase. Interestingly, at 42 kHz, the signal of (BA)_2_PbI_4_ is largely dominated by a broad isotropic chemical shift with a full width at half maximum of 12 kHz that does not decrease upon increasing the spinning frequency (see Fig. S6), and this likely stems from unresolved J- and dipole–dipole couplings to ^127^I.^74^ DFT calculations indeed show how the J-coupling is different in the two compounds, with stronger coupling present in the (BA)_2_PbI_4_ material due to different distortion of the Pb–I octahedra (see Tables S10 and S11). We stress that J-coupling is heavily affected by relativistic effects, and in this case, since we consider heavy Pb and I atoms, the results have more of a qualitative value, rather than quantitative. ^207^Pb ssNMR spectroscopy has been employed in several works to distinguish different coordination environments arising from halide mixtures,^43,68^ A-site cation composition,^20^ lowering of dimensionality (3D, 2D, 1D, 0D)^23,29^ and crystallographic position in multilayered RP phases.^6,28^ Here we show that ^207^Pb spectroscopy can also be conveniently applied to distinguish the different connectivities of 〈100〉- and 〈110〉-oriented perovskites by exploiting the strongly asymmetric coordination environment of the latter. Comparing our analysis with other lead iodide perovskites, we note that the ^207^Pb isotropic chemical shift and chemical shift anisotropy in (EDBE)PbI_4_ are comparable to those reported for the highly distorted Pb outer layers in the perovskite BA_2_MA_*n*−1_Pb_*n*_I_3*n*+1_ (MA = methylammonium) with *n* ≥ 3.^6^ We also probe a slightly higher isotropic chemical shift than the one reported for the 3 × 3 corrugated 〈110〉 perovskite (4NPEA)_2_PbI_4_ (with the signal located around 1500 ppm; 4NPEA = 4-nitrophenylethylammonium),^23^ further supporting our structural correlation.

As a complementary way to assess the local coordination environment, we performed saturation recovery measurements on ^207^Pb. Under MAS conditions, we probed very fast spin–lattice relaxation times *T*_1_ in the order of 30 ms for both compounds (see Fig. S7). This is consistent with the acceleration of longitudinal relaxation previously demonstrated in PbI_2_ and MAPbI_3_ caused by level-crossing and polarization exchange between lead and the adjacent ^127^I with quadrupolar spin.^74,75^ To obtain more representative values of the structural properties of the material, we further conducted the experiment under static conditions, with the results shown in Fig. 4d. These reveal that the increased asymmetry of the octahedral coordination in (EDBE)PbI_4_, arising from strong interactions between organic molecules and the inorganic cage,^76^ causes a faster ^207^Pb *T*_1_ relaxation (*T*_1_ = 0.0543 s) compared to (BA)_2_PbI_4_ (*T*_1_ = 0.351 s). The greater cation motion and variable orientations might additionally disrupt the symmetry of the inorganic framework, leading to local ^127^I positional disorder and faster inorganic layer dynamics in (EDBE)PbI_4_.^77^

The computation of the ^207^Pb NMR response is known to be very difficult and rarely goes beyond qualitative agreement with experiment^78–82^ and can be off by 1000 ppm.^83–85^ This is known to be true for molecular,^78^ cluster^79,80^ and periodic models.^84^ The discrepancy is due to different aspects: (i) the partial description of relativistic effects due to ^207^Pb nuclei; (ii) a limitation in computational resources available with a consequent hindering of accuracy; (iii) ^207^Pb chemical shielding can span values in the range of 10 000 ppm; as a result, even small relative errors result in large absolute deviations. However, the qualitative description of the system is correct with the chemical shielding (shift) of (BA)_2_PbI_4_ being lower (larger) than (EDBE)PbI_4_. Yet, comparing a dataset of simulations and experiment to obtain the chemical shift, we found a poor correlation between the two (*R*^2^ = 0.78, *m* = −0.51, see Table S9). As a result, the difference in chemical shift is qualitatively correct, with the chemical shielding of (BA)_2_PbI_4_ being above the one of (EDBE)PbI_4_ by 52 ppm. Interestingly, if SOC correction is completely omitted (instead of being applied to the pseudopotential), a better agreement with the experiment is achieved (see Table S12). This observation can be explained by dynamic effects or error cancellation.

## 3 Methods

The initial structures were obtained by X-ray diffraction and they had consequently been optimized using the Vienna *Ab initio* Simulation Package (VASP)^86^ to reduce each atomic force below the threshold of 0.05 eV Å^−1^, preserving the space group. The electronic problem has been solved using the projector-augmented-wave (PAW)^87^ formalism within the DFT. We employed the generalized gradient approximation (GGA) revised for the solid Perdew–Burke–Ernzerhof (PBEsol) exchange–correlation functional as implemented in VASP.

The planewave cut-off was set to 600 eV, while the first Brillouin zone has been sampled with a mesh of 4 × 4 × 1 for (BA)_2_PbI_4_ and 6 × 1 × 4 for (EDBE)PbI_4_. The dispersion forces have been described using the DFT-D3 (ref. 88) method including the Becke–Johnson damping function.^89^

The NMR tensors have been calculated for all the NMR-active species (namely ^1^H, ^13^C, ^15^N, ^127^I, and ^207^Pb) using the GIPAW method as implemented in the code CASTEP.^90^ The electronic parameters have been chosen to be consistent with the ones used for the geometrical optimization, but the planewave convergence threshold has been lowered to 10^−8^ eV. The *k*-points mesh has been reduced to have an interpoint distance smaller than 0.02 Å^−1^. This means a mesh of 6 × 7 × 2 for (BA)_2_PbI_4_ and 8 × 2 × 6 for (EDBE)PbI_4_. In contrast to VASP, CASTEP uses an on-the-fly-generated ultrasoft potential to describe the core-levels. While it is not possible to include a complete SOC correction directly in the calculation of the NMR response, a partial scalar correction can be included in the pseudopotential. In this work, we consider a scalar relativistic Koelling–Harmon approximation.^91^ A comparison between different relativistic corrections is presented in the SI. Since *in silico* simulation returns the absolute chemical shielding, it was necessary to calculate the chemical shift in order to compare the two sets of results, as explained in the SI.

ssNMR measurements were performed on a Bruker Avance NEO WB with a wide-bore magnet (89 mm) operating at a ^1^H frequency of 400 MHz. The chemical shift for all nuclei was referenced to the ^13^C signal of adamantane at 38.46 ppm.^65^^15^N chemical shifts are reported on the ammonia scale. A 1.9 mm cross polarization-magic angle spinning (CP-MAS) probe with a spinning frequency of up to 42 kHz was used for the characterization, employing 1.9 mm zirconia rotors with VESPEL turbines. One-dimensional ^13^C{^1^H} and ^15^N{^1^H} results were acquired through cross polarization by exciting the ^1^H nuclei and transferring the magnetization to the heteronucleus of interest. During the acquisition, heteronuclear decoupling was applied through the SPINAL64 sequence. The spin-rotation frequency was set to 25 kHz, 10 kHz and 42 kHz for ^13^C, ^15^N and ^1^H, respectively, while the probe temperature was controlled by setting the BCU chiller to 298 K.

The 90° pulse durations for ^1^H, ^15^N and ^13^C measurements were 1.66 µs, 3.33 µs and 2.94 µs, respectively. For ^13^C measurements, the contact time for CP was set to 4 ms for (BA)_2_PbI_4_ and 1.2 ms for (EDBE)PbI_4_. The ^13^C, ^15^N spin–lattice relaxation *T*_1_ was measured with the TORCHIA pulse sequence under MAS at 15 kHz and 10 kHz, respectively; for ^1^H, the inversion recovery pulse sequence was applied at 42 kHz. For ^15^N, contact times of 6 ms and 3 ms were used for (BA)_2_PbI_4_ and (EDBE)PbI_4_, respectively. ^207^Pb spectra were acquired with a Hahn-echo pulse sequence using a 90° pulse duration of 2.4 µs, relaxation delay of 0.1 s and accumulating 80 000 transients. Static measurements for ^207^Pb were performed by setting an echo delay of 12.4 µs, and increasing the number of scans up to 245 760. In the case of measurements at 20 kHz spin frequency, 500 000 accumulations were performed. The rotor spin frequency was set as specified in the text. Saturation recovery with an echo pulse sequence was used to measure the *T*_1_ spin–lattice relaxation of ^207^Pb, both under static conditions and at 20 kHz. The temperature was controlled with a Bruker BCU II chiller and varied as described in the text. Temperature calibration of the sample under magic angle spinning conditions was performed by measuring the spin–lattice relaxation time (*T*_1_) of ^79^Br in KBr *via* an inversion recovery experiment, as previously reported.^92^ Lineshape analysis and determination of the experimental chemical shift anisotropy and asymmetry was performed with the SOLA software package implemented in TopSpin.

## 4 Conclusions

We show that ssNMR spectroscopy is an effective characterization technique to probe the characteristic architecture of Ruddlesden–Popper (RP) and Dion–Jacobson (DJ) phases, providing distinctive features that allow the different local coordination environments of 〈100〉- and 〈110〉-oriented low-dimensional perovskites to be discerned. In the corrugated (EDBE)PbI_4_ DJ structure, the presence of two crystallographically independent sites arising from the characteristic supramolecular packing causes the peak splitting of both ^13^C and ^15^N signals in contrast with the (BA)_2_PbI_4_ phase with flat connectivity of the inorganic framework. In particular, static and spin–lattice relaxation measurements of ^15^N nuclei allow the different coordination environments arising from the corrugated pattern of the 〈110〉-oriented perovskite to be probed with great precision. ^207^Pb NMR spectroscopy can be conveniently used as a structural probe of the inorganic environment; notably, the distorted octahedral coordination of the zig–zag motif ((EDBE)PbI_4_) induces deshielding of ^207^Pb with a 541 ppm peak shift and significantly higher CSA and asymmetry compared to (BA)_2_PbI_4_. Fast MAS up to 42 kHz is necessary to minimize the chemical shift anisotropy interactions in the most distorted structure and yield high-resolution spectra; nevertheless, complementary static measurements should be performed to retrieve the full NMR tensor parameters, as well as to obtain ^207^Pb spin relaxation times representative of the perovskite structural properties. DFT calculations allow unequivocal assignment of all ^13^C and ^15^N spectral signals, although a 30 ppm underestimation is observed in the latter. For ^207^Pb simulations suffer of theoretical and technical limitations. Nonetheless, they provide a qualitative picture that is in agreement with the experimental data. Overall, this work provides a critical understanding of the ssNMR fingerprints of layered perovskites that will foster its use for the characterization of functional low-dimensional structures with more complex supramolecular assemblies.

## Author contributions

F. B. and F. L. performed the DFT calculations. N. S., G. M. and R. S. performed the solid-state NMR characterization. F. B., F. D. A. and D. C. coordinated and supervised the work. F. B. and D. C. conceived the scientific idea and drafted the manuscript. All authors contributed to the final version of the manuscript.

## Conflicts of interest

There are no conflicts of interest to declare.

## Supplementary Material

TA-013-D5TA02747K-s001

## Data Availability

Data for this article, including the experimental and computational data reported in the paper, are available at Zenodo at https://doi.org/10.5281/zenodo.16743235. Supplementary information is available with additional XRD and ssNMR measurements and computational details. See DOI: https://doi.org/10.1039/d5ta02747k.
