# Supplementary material for: Discerning two-dimensional metal halide perovskite moieties using solid-state NMR fingerprints
Source: J Mater Chem A Mater. 2025 Aug 5;13(39):33739–48. doi: 10.1039/d5ta02747k (PMC12418761; doi:10.1039/d5ta02747k)
Supplement: TA-013-D5TA02747K-s001 [file TA-013-D5TA02747K-s001.pdf]

Supplementary Information (SI) for Journal of Materials Chemistry A.
This journal is © The Royal Society of Chemistry 2025

Supporting Information of: Discerning two-dimensional metal halide perovskites moieties by solid state NMR fingerprints

Federico Brivio,[*,†] Nurgul Sarsembek,[¶] Giulia Martelli,[¶] Fabio Loprete,[¶] Rolando Scotillo,[¶] Filippo De Angelis,[‡,§,∥] and Daniele Cortecchia[*,¶,⊥]

†*Department of Physics and Astronomy "Galileo Galilei", University of Padova, Via Francesco Marzolo 8, 35131, Padova, Italy*

‡*Department of Chemistry, Biology and Biotechnology, University of Perugia, and Computational Laboratory for Hybrid/Organic Photovoltaics (CLHYO), Istituto CNR di Scienze e Tecnologie Chimiche Giulio Natta (CNR-SCITEC) Via Elce di Sotto 8, 06123, Perugia, Italy*

¶*Department of Industrial Chemistry Toso Montanari, University of Bologna, via Piero Gobetti 85, 40129 Bologna, Italy*

§*Institute of Energy Science and Technology (SIEST) Sungkyunkwan University (SKKU), Suwon 440-746, South Korea*

∥*Consorzio Interuniversitario Nazionale per la Scienza e Tecnologia dei Materiali (INSTM) - Unità di Ricerca (UdR) di Perugia, Via Giuseppe Giusti, 9, 50121 Firenze, Italy*

⊥*Center for Nanoscience and Technology, Istituto Italiano di Tecnologia, via Rubattino 81, 20134 Milano, Italy*

E-mail: federico.brivio@unipd.it; daniele.cortecchia2@unibo.it

1 Repository

The structure used for the study, the experimental data, along with a set of minimal input and scripts needed to reproduce the data reported in the paper is available on the online repository: https://gitlab.com/cippo1987/data_paper_pvk2d.

2 X-Ray Data

X-ray powder diffraction (XRPD) was performed on perovskite powders obtained from ground crystals using a BRUKER D8 ADVANCE with Bragg-Brentano geometry, Cu K_α radiation (λ= 1.54056 Å), step increment of 0.02 °C and 1 s of acquisition time. Figure S1 reports the comparison between the experimental X-Ray diffraction (XRD) data, and the simulated spectra of the structures derived from the previous.

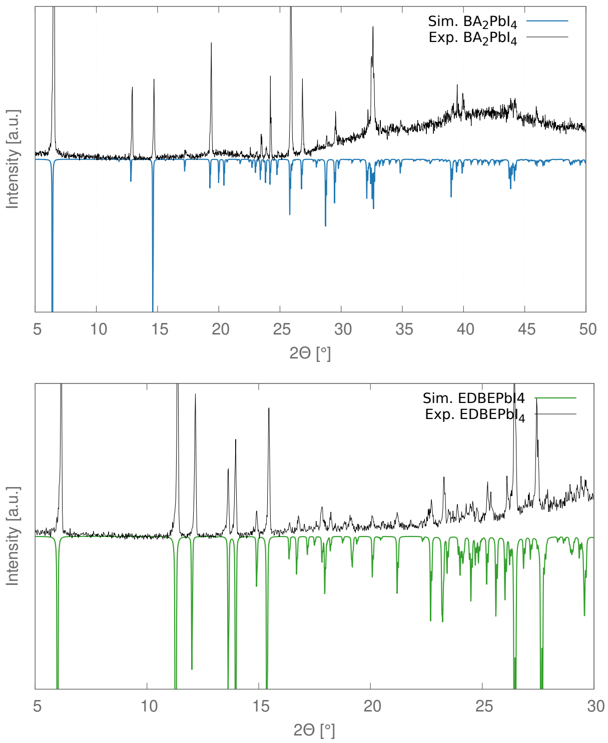

Figure S1: The black line in top (bottom) panel reports the experimental XRD for the $(BA)_2PbI_4$ sample. The blue (green) line is the simulated XRD data of the structural model derived for the $(BA)_2PbI_4$ ($(EDBE)PbI_4$) obtained from the experiment.

3 Local environments

Geometrical details of the NI environments and PbI_6 octahedra of the two compounds are reported in Table S2 and S1 of the crystallographic cell used for the computational part of the study. The distortion index for a polyhedra with n bond of average length $\bar{l}$, as described by Baur[1] is calculated by VESTA[2] as:

$$DI = \frac{1}{n}\sum_{i-=1}^{n}\frac{|l_i - \bar{l}|}{\bar{l}} \tag{S1}$$

The effective coordination number (Eff. CN) is calculated accordingly to Hoppe[3] by VESTA.

Table S1: Structural parameters of $(BA)_2PbI_4$ and $(EDBE)PbI_4$ PbI_6 octahedra. Both system have one symmetry independent Pb site. *d* is the average Pb−I bond length; *Vol* is the octahedra volume. *DI* is the distortion index; *Eff. CN* is the effective coordination number as defined by Hoppe.[3]

PbI_6	d [Å]	Vol [Å^3]	DI·10^{-3}	Eff. CN
$(BA)_2PbI_4$	3.172	42.45	6.5	5.99
$(EDBE)PbI_4$	3.189	42.87	25.7	5.73

Table S2: Structural parameters of $(BA)_2PbI_4$ and $(EDBE)PbI_4$ $N-I_n$ polyhedra. $(BA)_2PbI_4$ has only one environment of this type with n=5. A similar, but more distorted environment appears in $(EDBE)PbI_4$, which has also a second one with n=3. **d** is the average Pb−I bond length; *Vol* is the octahedra volume. *DI* is the distortion index; *Eff. CN* is the effective coordination number as defined by Hoppe. Max (Min) N−I column reports the longest (shorted) N−I bond.

$N-I_n$	d [Å]	Vol [Å^3]	DI[·10^{-3}]	Eff. CN	Max(N−I)[Å]	Min(N−I)[Å]
$(BA)_2PbI_4$ (n=5)	3.60	28.20	35.83	4.68	3.92	3.48
$(EDBE)PbI_4$ (n=5)	3.60	27.97	27.55	4.83	3.76	3.48
$(EDBE)PbI_4$ (n=3)	3.62	5.85	14.87	2.97	3.67	3.54

4 CP-MAS ssNMR experiment

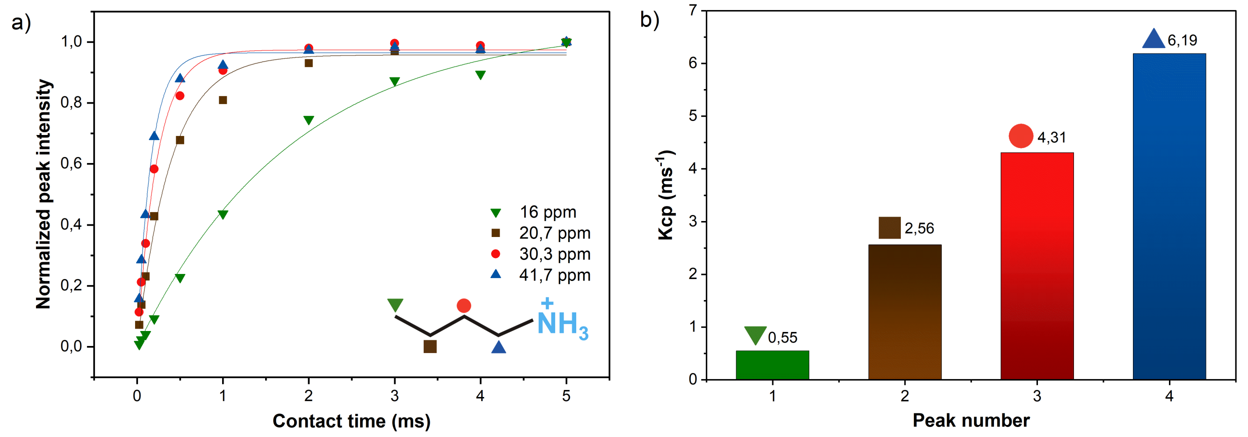

Figure S2: a) CP-MAS $^{13}C\{^{1}H\}$ solid-state NMR signal build-up curves for four distinct carbon sites in the BA cation (symbols), measured at 8 kHz MAS, with corresponding monoexponential fitting curves. The data represent the $^{1}H \rightarrow {}^{13}C$ magnetization transfer according to the equation: $I(t) = I_0\left(1 - e^{-Kcp}\right)$, where I(t) is the signal intensity at a given contact time t for each individual carbon, I_0 is the maximum intensity, and Kcp is the cross polarization transfer rate, which depends on dipole-dipole interactions between the nuclei. b) Calculated CP transfer rates (Kcp) for each carbon site in BA cation obtained from the fitting of build-up curves.

5 Intermolecular hydrogen bonding of $(EDBE)PbI_4$

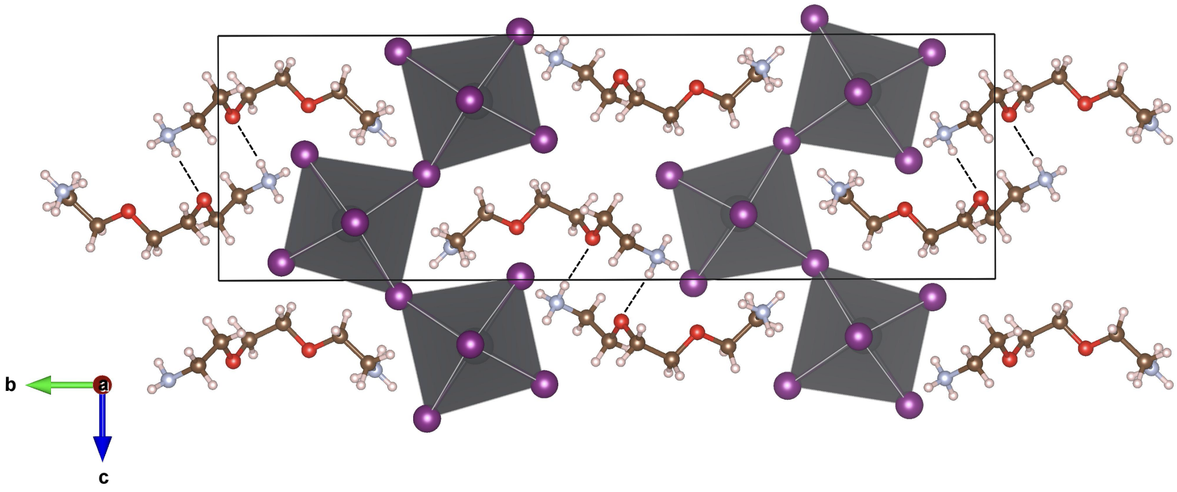

Figure S3: Schematic representation of $(EDBE)PbI_4$, dashed lines indicate hydrogen bonding between ammonium and ether groups within the organic spacer layers.

6 ^{1}H ssNMR spectra

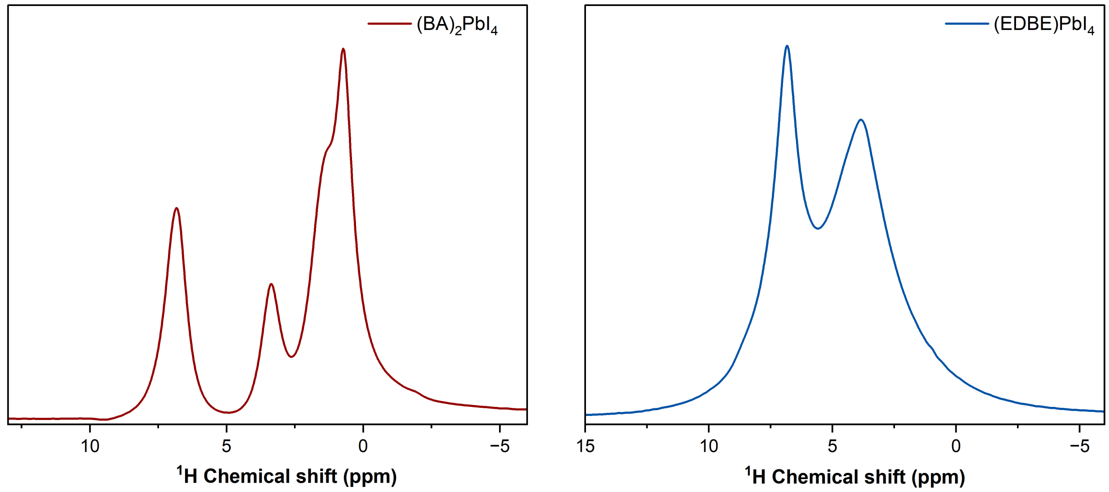

Figure S4: ^{1}H ssNMR spectra of $(BA)_2PbI_4$ (dark red) and $(EDBE)PbI_4$ (blue)

7 NMR results

Tables S5 and S6 report the values computed for the two systems using CASTEP with the PBESOL functional and the Koelling-Harmon SOC correction. With respect to the full shielding tensor the quantities reported are calculated as follows:

$$\sigma_{\text{iso}} = \frac{1}{3} Tr[\sigma_{ij}] \tag{S2}$$

$$\sigma_{\text{anis}} = \frac{\sigma_{zz} - (\sigma_{xx} + \sigma_{yy})}{2} \tag{S3}$$

$$\eta = \frac{(\sigma_{xx} + \sigma_{yy})}{(\sigma_{anis} + \sigma_{xx})} \tag{S4}$$

The chemical shift (δ) is obtained as described in the previous session. The definitions are done by assuming:

$$|\sigma_{yy} - \sigma_{iso}| \leq |\sigma_{xx} - \sigma_{iso}| \leq |\sigma_{zz} - \sigma_{iso}| \tag{S5}$$

Table S3: Experimental ssNMR peaks list and T_1 relaxation times for $(BA)_2PbI_4$.

$(BA)_2PbI_4$					
Peak max. [ppm]	T_1 [s]	Error [s]	Peak max. [ppm]	T_1 [s]	Error [s]
^{13}C			^{15}N		
41.77	6.84	0.34	50.5	12.8	0.69
30.32	5.51	0.18			
20.72	4.76	0.14			
15.96	2.49	0.07			
^{1}H			^{207}Pb (static)		
6.84	3.81	0.01	1103.79	0.351	0.002
3.33	3.90	0.02			
0.72	3.77	0.01			

Table S4: Experimental ssNMR peaks list and T_1 relaxation times for $(EDBE)PbI_4$.

$(EDBE)PbI_4$					
Peak max. [ppm]	T_1 [s]	Error [s]	Peak max. [ppm]	T_1 [s]	Error [s]
^{13}C			^{15}N		
72.01	3.28	0.14	46.31	4.06	0.46
68.90	0.509	0.01	41.72	16.7	1.41
68.01	3.13	0.16			
65.80	0.681	0.02			
43.70	0.393	0.01			
42.32	0.626	0.02			
^{1}H			^{207}Pb (static)		
6.81	2.05	0.01	1635.96	0.0515	0.0002
2.21	2.00	0.01			

Table S5: NMR Quantities calculated for the $(BA)_2PbI_4$ system. The Carbon atoms are listed in the same order (left to right) as they appear in the main text Figure 3c.

$(BA)_2PbI_4$	σ_{iso} [ppm]	σ_{anis} [ppm]	η	δiso [ppm]
C1	153	32	0.1124	17
C2	154	−12	0.7414	17
C3	141	32	0.6763	29
C4	135	−31	0.8234	36
N1	168	−13	0.7415	19
Pb	5593	−358	0.1721	3855

Table S6: NMR Quantities calculated for the $(EDBE)PbI_4$ system. The Carbon atoms are listed in the same order (left to right) as they appear in main text Figure 3d.

$(EDBE)PbI_4$	σ_{iso} [ppm]	σ_{anis} [ppm]	η	δiso [ppm]
C1	130	−21	0.8151	42
C2	102	64	0.1618	70
C3	104	72	0.3520	68
C4	99	60	0.4340	73
C5	104	59	0.2930	67
C6	132	−23	0.7410	39
N1	174	22	0.6045	12
N2	183	−22	0.6757	4
Pb	5692	228	0.6680	3804

8 ^{207}Pb NMR spectra temperature dependency

Figure S5 shows the temperature evolution of the ^{207}Pb ssNMR spectral response.

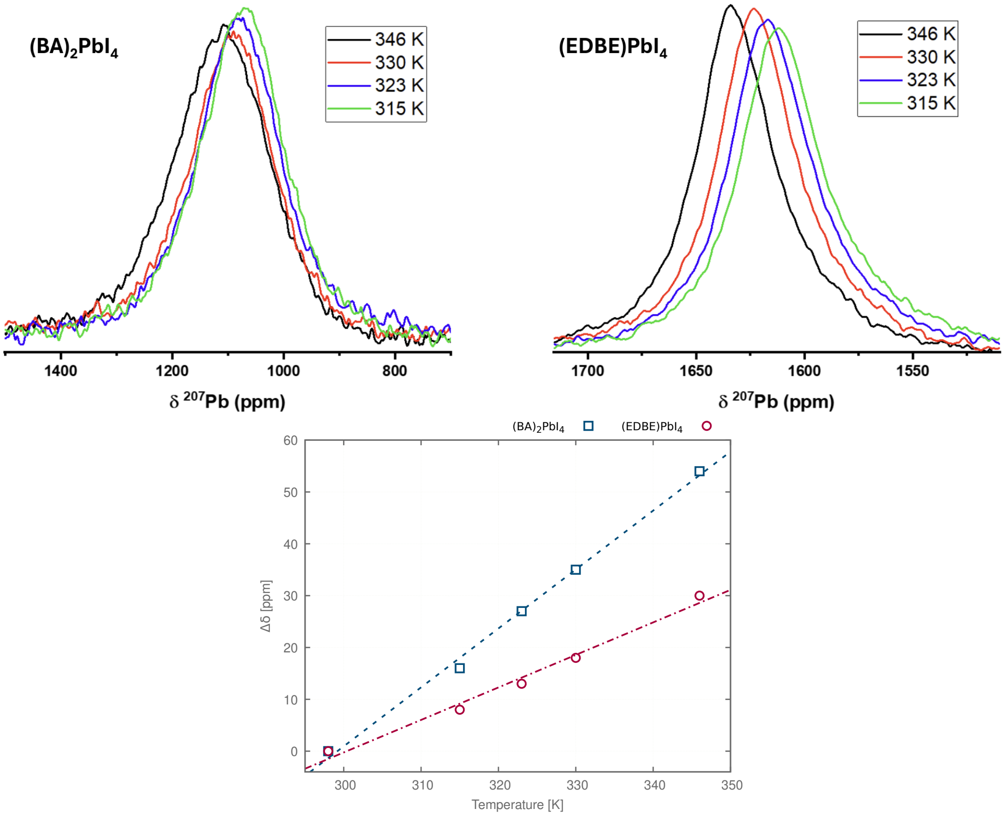

Figure S5: Top panel: shift of the ^{207}Pb NMR spectra in function of the temperature (MAS frequency of 42 KHz). Bottom panel: corresponding shift of the NMR peak in function of the temperature. The point at 298 K has been taken from a spectrum acquired at MAS frequency of 20 KHz. Data set refer to $(BA)_2PbI_4$ and $(EDBE)PbI_4$. The solid line are the result of the fitting of $y = a + bT$ where the slope b is 1.14 and 0.62 for $(BA)_2PbI_4$ and $(EDBE)PbI_4$, respectively.

9 NMR spinning frequency

Figure S6 reports the NMR spectra for $(BA)_2PbI_4$ and $(EDBE)PbI_4$ obtained at different spinning frequency of the rotor. It highlights the evolution of the sideband manifold of $(EDBE)PbI_4$, in contrast with $(BA)_2PbI_4$ where the sidebands are nearly absent even at low spinning speeds, indicating a small chemical shift anisotropy.

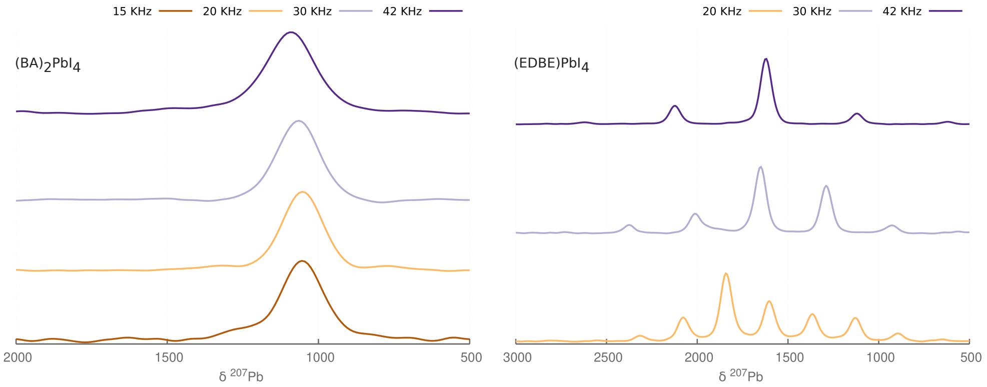

Figure S6: NMR spectra for $(BA)_2PbI_4$ and $(EDBE)PbI_4$ at different rotational speeds.

10 ^{207}Pb NMR relaxation time

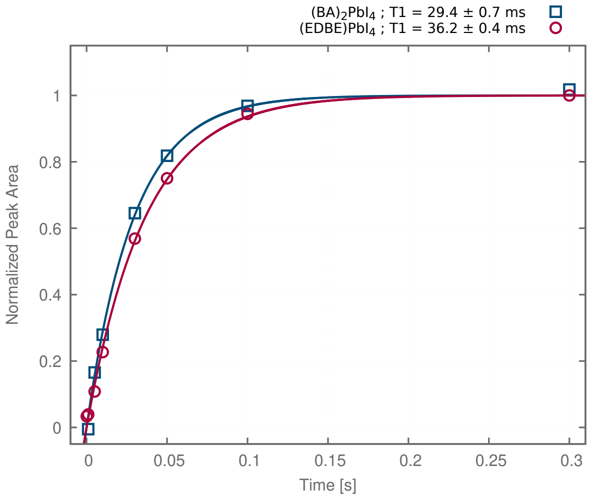

Figure S7: Comparison of the two different relaxation times between $(BA)_2PbI_4$ and $(EDBE)PbI_4$ measured employing saturation recovery with echo experiments under 42kHz MAS condition. Fitting to the experimental data was performed with the equation $y = I_0(1-e^{-\frac{t}{T_1}})$, where T_1 is 29.4 and 36.2 ms for $(BA)_2PbI_4$ and $(EDBE)PbI_4$, respectively.

11 Chemical shift calculations

DFT calculations return the chemical shielding of an atomic species. In principle, the chemical shift is obtained as a difference between the chemical shield of the species of interest and the same quantity of the reference material used in the experimental apparatus. However, in

general, it is preferable to obtain the chemical shift from a linear regression (see equation S6 between a set of experimental data and the correspondent theoretical results.[4] In this way it is possible to reduce the influence of eventual systematic errors. For a given atomic species i:

$$\sigma_i = c_1 + c_2\delta_i \tag{S6}$$

where the parameters c_1 and c_2 are obtained via linear least-squares fit against the experimental and theorethical data In an ideal case c_1 is the chemical shielding for the reference and c_2 is equal to -1.

11.0.1 ^{15}N chemical shift

Table S7 reports the experimental data and the compounds name taken from Ref.[4]

Table S7: Experimental chemical shift δ and theoretical chemical shielding σ used to obtain the ^{15}N chemical shift for our systems. The experimental data are taken from Ref.[4]

Name	δ_{iso} [ppm]	σ_{iso} [ppm]	Name	δ_{iso} [ppm]	σ_{iso} [ppm]
ASPARM03	75	100	GEHHIL	269	−75
	1	186		261	−60
CIMETD	213	−23	LCYSTN21	0	190
	150	39	LHISTD02	211	−23
	131	44		132	44
	57	121	LHISTD13	211	−24
	46	137		132	43
	44	143	LSERIN01	−4	192
LTYRHC10	8	178			

The previous data have a fitting coefficient R^2=0.9945, c_1=181.9 ppm, c_2=−0.9726

11.0.2 ^{13}C chemical shift

Table S8 reports the experimental data and the compounds name taken from Ref.[4]

Table S8: Experimental chemical shift δ and theoretical chemical shielding σ used to obtain the ^{13}C chemical shift for our systems. The experimental data are taken from Ref.[4]

Name	δ_{iso} [ppm]	σ_{iso} [ppm]	Name	δ_{iso} [ppm]	σ_{iso} [ppm]	Name	δ_{iso} [ppm]	σ_{iso} [ppm]
ADENOS12	155	18	INDMET	179	−12	LTYROS11	176	−7
	155	21		168	2		156	13
	149	27		157	13		131	39
	138	35		140	26		130	40
	120	51		138	32		123	50
	92	76		137	38		117	52
	85	85		134	38		117	54
	75	95		132	38		55	118
	71	99		131	40		36	135
	63	109		131	40	MEMANP11	100	68
ASPRM03	177	−3		129	42		72	99
	176	−9		127	44		72	100
	52	125		116	56		71	101
	36	138		113	57		65	108
FRUCTO02	100	68		113	61		59	114
	71	99		98	77		55	116
	69	104		55	117	MGALPY01	100	68
	67	105		28	146		73	98
	65	105		14	161		73	99
	65	106	LALNIN12	177	−9		70	105
GLTUAM01	177	−4		51	124		68	110
	174	−4		20	154		61	117
	54	122	LCYSTN21	175	−4		55	120
	29	145		54	119	MGLUCP11	101	67
	26	148		35	142		75	97
HXCAN09	170	4	LSERIN01	175	−7		75	97
	152	19		63	106		73	99
	133	40		56	119		72	101
	123	47	LSERMH10	176	−8		63.8	108
	121	51		62	109		57	114
	116	55		58	116	NAPHTA36	135	38
	116	56	LTHREO01	155	19		130	39
	24	149		114	60		129	40
				104	65		126	44
				−2	170		125	45

The previous data have a fitting coefficient R^2=0.9976, c_1=173.7 ppm, c_2=-1.020 .

11.0.3 ^{207}Pb chemical shift

Table S9 reports the experimental data taken from Ref.[5,6]

Table S9: Experimental chemical shift δ and theoretical chemical shielding σ used to obtain the ^{207}Pb chemical shift for our systems. The experimental data are taken from Ref.[5,6]

Name	δ_{iso} [ppm]	σ_{iso} [ppm]	Name	δ_{iso} [ppm]	σ_{iso} [ppm]
PbO_a	1939	5788	PbF_2	−2667	8217
PbO_b	1515	5649	$PbCl_2$	−1717	7571
Pb_3O_4	795	5971	$PbBr_2$	−979	7235
	−1105	6401	$PbSiO_3$	93	7049
Pb_2SnO_4	798	6405		−166	7206
	662	6443		−366	7281

The previous data have a fitting coefficient R^2=0.7819, c_1=6717 ppm, c_2=−0.5117

12 J-Coupling

The J-coupling calculations are not implemented in CASTEP to take in account Koelling-Harmon correction and are therefore they have been calculated with a scalar ZORA approach (see the *Spin-Orbital coupling* section 13).

Table S10: J-coupling for $(BA)_2PbI_4$ Iodine atoms. The Iodine atoms appear in the same order that can be found in the repository structure. For each atom is reported the reduced coupling constant K as output by CASTEP in the second and fifth column and the J coupling in HZ in the third and sixth column. The J coupling is obtained accordingly to $J = \frac{\hbar \gamma_I^2 K}{2\pi}$ where γ_I is the gyromagnetic constant of I = 5.37937 $\cdot 10^7$ [rad]$\cdot$Hz$\cdot$T^{-1}.

#I	K [$\cdot 10^{19}$kg m^{-2} s^{-2} A^{-2}]	J_{iso} [Hz]	#I	K [$\cdot 10^{19}$kg m^{-2} s^{-2} A^{-2}]	J_{iso} [Hz]
1	1526.14	741.23	9	3708.11	1800.99
2	1484.87	721.19	10	3720.84	1807.17
3	-0.80	-0.39	11	-0.27	-0.13
4	0.43	0.21	12	-8.43	-4.09
5	-12.98	-6.30	13	-1.86	-0.90
6	1.49	0.72	14	-1.90	-0.92
7	-48.39	-23.50	15	3780.66	1836.23
8	-48.85	-23.73	16	3857.50	1873.55

Table S11: J-coupling for (EDBE)PbI_4 Iodine atoms. The Iodine atoms appear in the same order that can be found in the repository structure. For each atom is reported the reduced coupling constant K as output by CASTEP in the second and fifth column and the J coupling in HZ in the third and sixth column. The J coupling is obtained accordingly to $J = \frac{\hbar\gamma_I^2 K}{2\pi}$ where γ_I is the gyromagnetic constant of I = 5.37937 $\cdot 10^7$ [rad]$\cdot$Hz$\cdot$T^{-1}.

#I	K [$\cdot 10^{19}$kg m^{-2} s^{-2} A^{-2}]	J_{iso} [Hz]	#I	K [$\cdot 10^{19}$kg m^{-2} s^{-2} A^{-2}]	J_{iso} [Hz]
1	756.59	367.47	9	-44.60	-21.66
2	-0.29	-0.14	10	6.24	3.03
3	-0.06	-0.03	11	1.26	0.61
4	380.00	184.56	12	295.48	143.51
5	3111.32	1511.14	13	9415.20	4572.87
6	-0.11	-0.05	14	0.74	0.36
7	0.25	0.12	15	0.13	0.06
8	3377.06	1640.20	16	419.58	203.79

13 Spin-Orbital coupling

CASTEP considers a scalar correction to the spin-orbital coupling effect only in the pseudopotentials.

We tested three different cases: the lack of correction (Non relativistic), and two scalar correction:(i) zeroth order regular approximation (ZORA) and (ii) the Koelling-Harmon (KH) method.

We can see that the inclusion of a scalar correction affects significantly the chemical shielding of the ^{207}Pb atoms.

We would like to stress that the difference between the two signal is opposite in sign if chemical shieldings instead of chemical shifts are considered. In light of this we can observe how the inclusion of SOC correction is not improving the description of the experimental results.

Table S12: Chemical shift (δ) and shielding (σ) in ppm for the two different materials. We considered no SOC correction (Non Relativistic - NR), Zora and Koelling-Harmon (KH) scalar SOC corrections. The last line reports the difference between the peaks of the two compounds. For the theoretical data, this difference is opposite in sign to what should be observed experimentally.

^{207}Pb [ppm]	δ(Exp.)	σ(NR)	σ(ZORA)	σ(KH)
$(BA)_2PbI_4$	1070	5628	6918	5593
$(EDBE)PbI_4$	1611	6522	7007	5692
Δ	541	894	89	99

References

(1) Baur, W. The geometry of polyhedral distortions. Predictive relationships for the phosphate group. Acta Crystallographica Section B: Structural Crystallography and Crystal Chemistry **1974**, 30, 1195–1215.

(2) Momma, K.; Izumi, F. VESTA 3 for three-dimensional visualization of crystal, volumetric and morphology data. Journal of applied crystallography **2011**, 44, 1272–1276.

(3) Hoppe, R.; Voigt, S.; Glaum, H.; Kissel, J.; Müller, H. P.; Bernet, K. A new route to charge distributions in ionic solids. Journal of the Less Common Metals **1989**, 156, 105–122.

(4) Hartman, J. D.; Kudla, R. A.; Day, G. M.; Mueller, L. J.; Beran, G. J. Benchmark fragment-based 1 H, 13 C, 15 N and 17 O chemical shift predictions in molecular crystals. Physical Chemistry Chemical Physics **2016**, 18, 21686–21709.

(5) Fayon, F.; Farnan, I.; Bessada, C.; Coutures, J.; Massiot, D.; Coutures, J. Empirical correlations between 207Pb NMR chemical shifts and structure in solids. Journal of the American Chemical Society **1997**, 119, 6837–6843.

(6) Alkan, F.; Dybowski, C. Chemical-shift tensors of heavy nuclei in network solids:

a DFT/ZORA investigation of 207 Pb chemical-shift tensors using the bond-valence method. Physical Chemistry Chemical Physics **2015**, 17, 25014–25026.
